# Self-injurious behavior in individuals with intellectual and developmental disabilities: An interdisciplinary family systems review

**DOI:** 10.1111/jftr.12548

**Published:** 2023-12-13

**Authors:** Caroline L. Roberts, Frank Symons

**Affiliations:** Department of Educational Psychology, University of Minnesota, Minneapolis, Minnesota, USA

**Keywords:** disability-related studies, families and psychopathology, families with children who have disabilities, family and mental illness, family systems theory, interdisciplinary, mental retardation/developmental disabilities, self-injurious behavior, special needs children

## Abstract

This conceptual review paper takes an interdisciplinary approach to the study of self-injury in families. The overall goal is to begin integrating siloed bodies of knowledge from empirical work based on findings from individuals with and without intellectual and developmental disabilities and self-injury. The research literature on self-injury and family-level variables is reviewed, including dyadic and individual-level variables with potential bidirectional impact on the family. Then, opportunities for knowledge translation are explored with respect to the pragmatic goal of developing family-level interventions for self-injurious behavior in individuals with intellectual and developmental disabilities. Four opportunities for knowledge translation are highlighted: family patterns, parenting behavior, attachment relationships, and emotional experience.

And it’s not like you can ask the medical community or the educational community or the therapy community, none of them have the answers. They just all will try to help from their individual discipline, but they are not the family who sees the whole picture and who is trying to have a semi-decent life day to day.

Roberts et al., 2023, p. 13

Self-injury affects the entire family and is, in turn, affected by the family system.

Kissil, 2011, p. 313

Self-injurious behavior (SIB) associated with intellectual and developmental disability (IDD) is a complex clinical and scientific problem with potentially devastating impacts on quality of life (Bradley et al., 2018). For families and caregivers, SIB disrupts family life, lowers well-being, and creates chronic stress at multiple levels of the family system (Griffith & Hastings, 2014; Rojahn et al., 2008). Challenging behaviors like SIB deplete economic resources in the educational and social service systems and professionals treating or managing SIB, such as teachers and direct care staff, experience negative psychosocial effects as well (Mossman et al., 2002). There is not a clear etiology or known developmental trajectory of SIB in IDD, despite recent advances in biobehavioral treatment options (Furniss & Biswas, 2020). Even with these advances in treatment, “there is little evidence that the scientific community has effectively reduced the long-term burden of SIB on families and society” (Heath et al., 2015, p. 799). This long-term burden is clear in firsthand accounts from family caregivers, who describe the challenge of “trying to have a semi-decent life day to day” while living with chronic SIB in IDD (Roberts et al., 2023).

## AIMS AND APPROACH

The overall aim of this conceptual review is to describe the issue of SIB in IDD from a family systems lens. By selectively reviewing scientific and clinical literatures from multiple points of view, we engage the family science community by introducing the problem of SIB in IDD and creating a theoretical context for the development of interventions for family practitioners.

We engage the family science community because there is very little research specific to SIB and IDD and families. When SIB in IDD is studied, it is rarely at the family level; rather, it tends to be discussed at the individual or dyadic level (e.g., behavioral contingencies between a parent and a child or a behavior therapist and a child). Yet, self-injury onset is often in childhood or adolescence, and these are developmental stages when the family context is especially salient (Cipriano et al., 2017; Heath et al., 2015).

We frame this review in a family systems theoretical lens in order to capture the bidirectional influence of SIB on the individual and their family. According to family systems theory, the family is a single, interdependent emotional unit, made up of multiple dynamic relational subsystems (Bowen, 1966). The behavior of each individual in the family influences the family system and vice versa. When a child or adolescent is engaging in a maladaptive behavior like self-injury, it impacts other family members and the family system as a whole. Aspects of the family system and other family members are likely impacting the child or adolescent’s engagement in self-injury.

Our strategy to address the lack of research on SIB in IDD at the family level is to draw from the parallel literature of family-level research on nonsuicidal self-injury (NSSI) in individuals without IDD. We are making the choice to discuss potential application of NSSI research to SIB because there is a stronger tradition of family dynamics research in the study of NSSI. Most notably, research-based family-level interventions for NSSI exist while they do not for SIB. Important distinctions notwithstanding (e.g., topographical patterns and self-report), we discuss how the research findings on NSSI in families can inform conceptualizations, practice, and future research on SIB in IDD in families. We combine multiple academic disciplinary perspectives in pursuit of possible new ways to consider SIB in IDD by taking an exploratory, interdisciplinary approach drawing from the fields of psychiatry, behavioral psychology, applied behavior analysis, and family science to answer the primary research question: In children and adolescents with IDD, how does SIB impact the family and how does the family impact SIB?

## SELF-INJURY: DEFINITION, CLASSIFICATION, AND TERMINOLOGY

Self-injury is defined as “the deliberate destruction of one’s own bodily tissue…for reasons not socially sanctioned” (Cipriano et al., 2017, p. 1). This definition excludes socially sanctioned behaviors like tattoos or piercings and includes various forms or topographies such as cutting, burning, hitting oneself, headbanging, scratching, and skin picking.

Typically, two distinctions are made in the classification of self-injury: whether the behavior has suicidal intent, and whether the individual does or does not have IDD. This review focuses on self-injury without suicidal intent. The second distinction, whether an individual does or does not have IDD, has profoundly shaped the way self-injury is researched and treated. When an individual does not have IDD, self-injury without suicidal intent is most often referred to as *nonsuicidal self*-*injury* or NSSI. NSSI is typically under the disciplinary purview of psychiatry and understood with a neuropsychological lens as a manifestation of an underlying psychiatric disorder and/or as a maladaptive affect regulation strategy.

When an individual engages in self-injury and has an intellectual and/or developmental disability, self-injury is most often referred to as *self*-*injurious behavior* or SIB. SIB is typically under the disciplinary purview of behavioral psychology, specifically applied behavioral analysis (ABA). It is most often understood from a behavioral lens, emphasizing the roles of reinforcement contingencies and learned behavior (Dimian & Symons, 2022).

While terminology is not always consistent in the research literature, for clarity NSSI is used in this paper to discuss self-injury in individuals who do not have a diagnosed intellectual or developmental disability, and SIB is used to discuss self-injury in individuals who have one or more diagnosed intellectual and/or developmental disabilities. The suicidality distinction is also not always consistently made in the research literature; when possible, research which does not clearly distinguish suicidal intention, thoughts, or actions with self-injury that does not have suicidal intent is identified as such. As will be discussed later in the paper, key differences between SIB and NSSI include differences of form, concealability, and ability to self-report.

Other terms in use in the research literature include *self*-*harm*, *self*-*mutilation*, *self*-*abuse*, *self*-*assault*, *autoaggression, self*-*destructive behavior, stereotypic SIB*, *challenging behavior*, *problem behavior*, or *aberrant behavior* (Yates, 2004). Depending on whether the authors discuss the behavior in the context of individuals with or without IDD, results of the studies and work reviewed are discussed as SIB or NSSI, respectively. The broad term *self-injury* is used here when referring to both populations, those with and without IDD.

## LITERATURE REVIEW

We conducted a search of major relevant databases (i.e., PubMed, PsycINFO) with the aforementioned terms to describe self-injury as well as search terms related to family-level factors and interventions (i.e., “family,” “parent child,” and “family therapy”). As this is a selective conceptual review rather than a systematic review, we read the literature with the exploratory goal of knowledge translation rather than systematic digestion of all literature available. Toward this end, we particularly draw from several review papers on NSSI and families to characterize this literature (i.e., Baetens et al., 2014; Buckmaster et al., 2019, 2021, Fortune et al., 2016; Yates, 2004; similar review papers do not exist for SIB and families).

Relevant research domains on families and the issue of self-injury are considered in three major sections: family-level variables, dyadic variables, and individual-level variables with potential bidirectional influence on the family. Dyadic variables refer to variables within two-person subsystems of the family (parent–child, parent–parent, and sibling–sibling). Within each section, specific variables are discussed as correlates with self-injury or as promotive, protective, or risk factors for self-injury (when the authors identify the variables with these terms). According to theories of resilience in human development, a promotive factor is “associated with adaptive success independent of risk or adversity level” (e.g., internal capabilities, external resources) while a protective factor is one which may “mitigate risk or bolster recovery in families experiencing major adversities” (Masten & Monn, 2015, pp. 9, 12, e.g., emotional attunement).

Because of the limited research specific to SIB in IDD in the domains described above, the discussion of each variable begins with an overview of research in NSSI, followed by research on SIB when available. When research specific to SIB is not available, there is instead a discussion of research on the broader category of challenging behavior in children and adolescents with IDD. The category of *challenging behavior* is commonly used in the field of intellectual and developmental disabilities and typically includes SIB as well as aggression, destructiveness, inappropriate sexual behavior, antisocial behavior, disturbed sleep, and over-activity (James, 2013). When neither research on SIB nor challenging behavior is available, this is acknowledged as a research gap.

### Family-level variables

#### Family cohesion

Family cohesion has been identified as a protective factor by family stress researchers (Masten & Monn, 2015). The construct of cohesion, sometimes referred to as closeness or togetherness, comes from the Circumplex Model of Family Systems (Olson, 2000). The Circumplex Model posits the three primary dimensions of a family system as cohesion, flexibility, and communication (Olson, 2000). Cohesion is defined as “the emotional bonding that family members have toward one another” (Olson, 2000, p. 145) and has four levels: disengaged, separated, connected, and enmeshed; disengaged or enmeshed levels of cohesion are generally considered dysfunctional (Olson, 2000). A cohesive family promotes adaptation to a crisis or stressor such as self-injury at both the family and individual levels.

There are documented associations between NSSI in adolescents and dysfunctional levels of family cohesion (Buckmaster et al., 2021). In disengaged families, Buckmaster et al. (2021) described a family culture wherein children are expected to be “seen and not heard;” the theme of “self-harm as evidence of madness” (pp. 673–674). On the other end of the cohesion continuum, enmeshed (or sometimes called overinvolved) families were characterized by hypervigilance, overbearance, and the sentiment: “we’re such a close family that if they were in pain, we were in pain” (Buckmaster et al., p. 674). The association between NSSI and cohesion is robust and has been both qualitatively and quantitatively identified as an important variable for family-level intervention (Buckmaster et al., 2019, 2021; Fortune et al., 2016). Elements of family cohesion are often a target of family-level interventions for NSSI, even if the term *cohesion* is not necessarily used in intervention descriptions or therapeutic practice. For example, emotionally-focused family therapy directly addresses the emotional bonding between family members through changes such as increasing parent responsiveness (Schade, 2013).

Lanfranchi and Vianello (2012) found no statistically significant differences in family cohesion by diagnostic groups that are or are not associated with increased challenging behavior and SIB (Down syndrome, Williams syndrome, fragile X syndrome, and Prader-Willi syndrome). Despite a lack of research specific to SIB or challenging behavior and family cohesion, cohesion is associated with several other variables relevant to families of children with IDD, including reduced parenting stress, greater family satisfaction, and greater emotion-oriented coping (Taylor et al., 2023). Siblings tend to have higher relationship quality in cohesive families (Taylor et al., 2023).

#### Family communication

Family communication is a second dimension of the Circumplex Model (Olson, 2000). Olson (2000) describes communication as the *facilitating dimension*, which is “critical for facilitating movement on the other two dimensions” in family therapy (p. 149). Important specific communication skills include speaking skills, listening skills, self-disclosure, clarity, respect, and regard (Olson, 2000). Family communication matters because effective communication contributes to both an individual and a collective sense of resilience (Theiss, 2018).

Wester and King (2018) explored associations between NSSI and family communication patterns in a population of undergraduate students. They measured family communication patterns with the Revised Family Communication Pattern (Ritchie & Fitzpatrick, 1990), which consists of a subscale measuring a family’s degree of open dialogue (the *Conversation Orientation Subscale*) and a subscale measuring a family’s value of agreement over dissenting individual opinions (the *Conformity Orientation Subscale*). Family communication patterns were not associated with current NSSI in the undergraduates, but they were associated with lifetime NSSI, particularly the *Conversation Orientation Subscale* (Wester & King, 2018). Individuals from a family with a conversational orientation toward more open dialogue were less likely to have past NSSI (Wester & King, 2018). Family communication relates to the skill of self-disclosure as identified in the communication dimension of the Circumplex Model. NSSI is often a hidden behavior; these studies support the possibility that its presence, particularly if hidden, indicates a lack of open dialogue and self-disclosure in a family system.

Research on communication and SIB in IDD usually refers to the communication skills of the child or adolescent with IDD; there is an association between lower communication abilities and greater likelihood of engaging in SIB (McClintock et al., 2003). In terms of the family-level construct of communication, however, there is no research specific to how families experiencing SIB in their child with IDD communicate. More generally, however, there is some research on family communication patterns in families of a child with IDD (regardless of the presence of SIB or challenging behavior). For example, in a study in South Africa, Greeff and Nolting (2013) acknowledged the increased stress of parenting a child with IDD and the family adaptation required. They conceptualized adaptation according to resilience theory and analyzed the adaptive processes of 46 families through both quantitative and qualitative methods. Their analysis found that positive patterns of family communication were the most significant predictor of family adaptation, suggesting family communication is important in families with and without IDD.

#### Family flexibility

Family flexibility, the final dimension of the Circumplex Model, refers to how adaptive the family is via “change in (the family’s) leadership, role relationships, and relationships roles” (Olson, 2000, p. 147). Flexibility or its converse, rigidity, is a common construct in the literature on parent–child interactions, and there may be varying and dynamic degrees of flexibility in each subsystem and relational role in a family. Like cohesion, flexibility is measured on four levels (rigid, structured, flexible, chaotic) and either extreme, too rigid or too flexible, is considered potentially dysfunctional, particularly when a family experiences a high degree of stress (Olson, 2000).

In adults, Buckmaster et al. (2019) recommended promoting flexibility as a best practice in therapy for families experiencing NSSI. In adolescents, Fortune et al. (2016) also identified the ability to negotiate change as a protective factor in NSSI, though they referred to the construct as *adaptability* and did not distinguish between suicidality and NSSI in their review.

In a 3-year longitudinal study of families of adolescents with autism spectrum disorder, Baker et al. (2011) found that family adaptability predicted change in both maternal depression and child challenging behavior. They found these effects were maintained regardless of whether the adolescent had a co-occurring intellectual disability and concluded “the benefit of family adaptability may not depend upon children’s functional abilities” (Baker et al., p. 605). While SIB was included in their measure of challenging behavior, the data were not disaggregated by type of challenging behavior, so it is unclear if the role of family flexibility differs when a child is engaging in SIB compared with other challenging behaviors. However, this study supports the notion that family flexibility is an important variable for family-level intervention in SIB as it is in NSSI.

#### Family functioning

In the NSSI literature, some studies use a single measure to describe family functioning while others use *functioning* as an umbrella term for several measures indicating overall health of the family system. Given this inconsistency, construct measurement information is provided alongside results in this section.

Baetens et al. (2014) found no statistically significant associations between family functioning and NSSI in preadolescents (specifically, 12-year-olds living in Belgium). They measured family functioning with the Vragenlijst Gezinsproblemen, a Dutch language adaptation of the Family Assessment Measure, a single measure completed by parent-report that contains four subscales of support and communication, commitment, security, and couple relationship.

Cassels et al. (2018) measured family functioning with the McMaster Family Assessment Device General Functioning Subscale; this is a 14-item self-report subscale used to describe “quality of family relationships” (p. 883). They found an association between poorer family functioning and the onset of NSSI between the ages of 14 and 17 (Cassels et al., 2018). Further, family functioning mediated an association between childhood family adversity and new onset NSSI in teens.

In Buckmaster et al.’s (2019) systematic review, *family functioning* was an umbrella term and subtheme title under the broader theme of family unit factors. This subtheme described information from four studies, reporting information on family dynamics, family cohesion, flexibility, level of contact, and perceptions of support, and was summarized with the statement “self-harm was found to be related to poor family functioning” (Buckmaster et al., p. 537). The inconsistency in findings could be due to the variability in how the construct of family functioning is defined and measured, making it difficult to draw definitive conclusions about the role of family functioning in NSSI.

In individuals with IDD, “only recently have researchers begun to consider the influence of the family environment on the trajectory of child behavior problems over time” (Furniss & Biswas, 2020, p. 38). There are no studies specifically investigating family functioning and SIB in IDD at this time. Though not specific to SIB, there were studies available on individuals with autism spectrum disorder and/or challenging behavior and similar constructs such as *family well-being* (Tint & Weiss, 2016) and *home atmosphere* (Totsika et al., 2014). Totsika et al.’s (2014) measure of home atmosphere (also referred to as *home chaos*) was assessed with three items from the *Confusion, Hubbub, and Order Scale* (CHAOS). These items were “disorganized house, you can’t hear yourself think, and calm atmosphere at home” (Totsika et al., 2014, p. 426). They found no long-term associations between child challenging behavior and home atmosphere, though they did find an association between challenging behavior and relationship quality (Totsika et al., 2014). Again, it is difficult to draw conclusions about family functioning and SIB due to underdeveloped research in this area, particularly as it is unclear the degree to which the construct of *home atmosphere* overlaps with the construct of family functioning as it is variously defined in the NSSI literature.

#### Conflict, abuse, and trauma and the family

There is a robust literature connecting NSSI with negative relational risk factors such as conflict, abuse, and trauma. A systematic review of interpersonal processes and NSSI identified exposure to harmful relational patterns as a risk factor for engaging in NSSI (Peel-Wainwright et al., 2021). In particular, experiencing childhood sexual abuse was strongly associated with engagement in NSSI and mediated by post-traumatic stress symptoms (Weierich & Nock, 2008). Yates (2004) describes a pathway from childhood trauma to the development of NSSI within a developmental psychopathology framework. Her model presents a traumagenic hypothesis for NSSI in which a child’s experience of trauma in the caregiving relationship “compromises the quality of the individual’s adaptation at motivational, attitudinal, instrumental, emotional, and/or relational levels of competence,” ultimately leading to NSSI (Yates, 2004, p. 54).

Conflict in family relationships was identified as a risk factor for self-injury in a systematic review of youth from low- and middle-income countries, including China, India, and Turkey (Aggarwal et al., 2017). Crowell et al. (2013) coded mother–child interactions with a microanalytic coding scheme and found that adolescents who self-injured were more likely to escalate conflict with their mothers; they interpreted this finding as evidence that emotional invalidation and conflict escalation in the parent–child relationship are risk factors for self-injury. Cassels et al. (2018) found poor family functioning mediated the association between experiencing childhood adversity and engaging in NSSI as an adolescent. From an ecological lens, there is also research indicating that family support or dysfunction can influence the impact of abuse from outside the family. Hooven et al. (2012) found that family dysfunction or support moderated the effect of peer victimization on emotional regulation.

Overall, it seems clear that experiencing conflict, abuse, and trauma in childhood and adolescence increases the likelihood of engaging in NSSI. When these negative experiences are originating from outside the family, the family can either increase or decrease the likelihood of engaging in NSSI, depending on how the family is functioning and whether the adolescent is receiving family support.

There is less literature available on conflict, abuse, and trauma in families with IDD as it relates to challenging behavior like SIB. Totsika et al. (2014) found an association between increased parent–child conflict and increased child behavior problems at age 3 as well as (in the same cohort) at age 5, but their measure of challenging behavior did not include SIB (*Strengths and Difficulties Questionnaire*; Goodman, 1997).

Individuals with IDD are more likely to experience trauma and abuse than individuals without IDD (Emerson & Hatton, 2007; Stalker & McArthur, 2012). Disability researchers are currently grappling with issues related to trauma in individuals with IDD, such as whether the definition of post-traumatic stress disorder used in individuals without IDD applies in the same way to individuals with IDD, or how to assess trauma exposure in individuals with communication difficulties (Wigham & Emerson, 2015). However, what is relatively clear is the association between experiencing trauma and engaging in challenging behaviors (Wigham & Emerson, 2015), though it is not clear if SIB differs from other challenging behaviors in this context. Given this association with challenging behavior broadly and the robust state of the literature on trauma and NSSI (i.e., Yates, 2004), it is worthwhile for the emerging conversation on trauma and individuals with IDD to consider the roles of SIB and the family context.

#### Socioeconomic status

Lower family socioeconomic status is consistently related to higher rates of adolescent engagement in NSSI, variously referred to in the research literature as family socioeconomic disadvantage, socioeconomic adversity, lower income, household economics, or socioeconomic status (Baetens et al., 2014; Fortune et al., 2016). However, in the Fortune et al. (2016) study, accounting for related factors like family history of mental illness made the effect statistically nonsignificant, suggesting a complexity beyond a linear relationship between SES and NSSI (however, this research did not separate findings by suicidality). More research is needed to extricate the complexity of the relationship between family socioeconomic status and adolescent engagement in NSSI.

Socioeconomic status may be a risk factor for the development of SIB in IDD, but this notion needs more research as well (Furniss & Biswas, 2020). Baghdadli et al. (2003) did not find a statistically significant association between presence of SIB in French children with autism and parent social class, yet other studies have suggested associations between SIB in children with autism and other variables associated with SES, such as maternal education and cigarette smoking during pregnancy (Soke et al., 2016).

#### Family structure

Some researchers have investigated types of changes that families commonly experience and how a family’s reaction to them might influence a family member’s NSSI. A change in family structure emerged as a theme in a study of Latina adolescent girls who self-injure (Gulbas et al., 2015). In this qualitative study, girls and women frequently reported experiencing loss related to changes in family structure (i.e., divorce, death, or migration; 2015).

Single-parent families and families with divorced parents are associated with the presence of NSSI in adolescents, as is having divorced parents with new partners (Aggarwal et al., 2017; Nixon et al., 2008). Ponnet et al. (2005) found an interaction effect with child sex: boys in single-parent families and girls in remarried families report greater NSSI than either boys or girls from two-parent, first-marriage families. However, it is important to acknowledge that certain family structures do not *cause* adolescent engagement in NSSI, nor do these associations fully represent the complexity of a family.

There is little research on family structure and challenging behaviors including SIB in IDD. What does exist tends to relate to parenting stress. For example, single mothers of children with autism are more vulnerable to depression than partnered parents of children with IDD (not including autism) or children without IDD or autism (Olsson & Hwang, 2001). Olsson and Hwang (2001) suggest the higher likelihood of challenging behavior in autism (and resulting stress) as a potential explanation for this effect.

A related topic that is more researched in the IDD population is the residential placement of a child with IDD and SIB outside the family home (e.g., a group home or institutional setting). The prevalence of SIB increases in more restrictive environments (from independent living to institutionalization, with the family home as the second least-restrictive environment after independent living; Rojahn et al., 2008). Chadwick et al. (2008) found that lack of continuity in parental care (in some cases due to residential placement) was associated with increased overall behavior problems but not statistically significantly associated with SIB specifically. However, they encourage caution in interpretation of this finding because it is unknown if disruptions in parental care lead to challenging behaviors or if challenging behaviors lead to disruptions in parental care (or both). More research on SIB in IDD as it relates to family structure is needed, particularly with respect to the role of residential placement.

#### Genetic influence

Familial genetics plays an important role in the NSSI research in terms of an individual’s family history of mental illness. Several mental illnesses with known genetic links are associated with NSSI, such as bipolar disorder (Heath et al., 2015), as well as personality disorders such as antisocial, narcissistic, histrionic, and borderline personality disorders (Fox et al., 2015). In addition, there is an emerging research area investigating genetic and epigenetic implications in NSSI, including gene–environment interactions (Kaess et al., 2021).

The role of family history from the genetic perspective is one of the only topic areas on family and SIB in IDD with significant available research literature. Specifically, this relates to the handful of syndromes with known genetic etiology and in which SIB frequently co-occurs: Lesch Nyhan syndrome, Cornelia de Lange syndrome, Rett syndrome, fragile X syndrome, and Smith Magenis syndrome are the most common (though these syndromes are still quite rare; Arron et al., 2011). Each of these syndromes has a known genetic etiology and associated occurrence of SIB, though the most common topographies differ and within-syndrome variability exists (Langthorne & McGill, 2008). Thus, the biological family impacts the presence of SIB through the contribution of genetic material in addition to environmental influence. However, how the genes implicated in each of these syndromes are related to the risk of developing SIB and their interaction with psychosocial variables is unclear (e.g., some boys with fragile X syndrome with a common genetic mutation and similar functional outcome level engage in SIB and some do not; Symons et al., 2010).

#### Overall summary of family-level variables and self-injury

With respect to supporting families experiencing SIB in IDD, overall, more research is needed, particularly on variables, which are potential intervention targets for family practitioners. It seems likely that the Circumplex Model of Family Functioning (Olson, 2000) would have similar utility in SIB as it does in NSSI, given research indicating that cohesion, communication, and flexibility are important variables for families of individuals with IDD (Baker et al., 2011; Greeff & Nolting, 2013; Taylor et al., 2023). In particular, family communication is foundational and critical in family-level interventions for NSSI, and this is likely to be true in SIB as well. Family functioning, in the current state of the research, requires greater attention to construct validity to be useful in clinical settings. Perhaps the breadth of the family functioning construct makes it less useful as an intervention target. Instead, more specific variables that are sometimes included within the construct of family functioning (e.g., cohesion, flexibility) should have increased focus.

More research on SIB in IDD as it relates to family structure is needed particularly with respect to the role of residential placement. As families usually have agency over if and how their child with IDD is placed outside the home (and transitions of this nature can be a major adjustment for families), this element of family structure may be especially important for family practitioners treating SIB to consider, particularly as families experiencing SIB in IDD may seek decision-making support.

Similarly, conflict, abuse, and trauma may be relevant for family practitioners, but this research area is significantly underdeveloped in SIB, especially in comparison with the depth of research available in NSSI. Variables such as socioeconomic status and genetics may be associated with SIB in IDD, but they are not malleable intervention targets for family practitioners. However, it is still important for family practitioners to consider how these factors may influence the family in the context of self-injury.

### Dyadic-level variables

According to family systems theoretical perspectives, dyadic relationships represent subsystems within the greater family system. In this review, the term *parent* may refer to primary caregivers other than biological parents, including adoptive parents or foster parents, though the majority of the research has been conducted with biological parents.

#### Parent–child

##### Attachment

In attachment theory, a child learns emotional self-regulation through secure attachment with a caregiver. When NSSI is present, this may represent a failure of self-regulation related to an insecure attachment (Kissil, 2011). A family systems perspective is crucial to assessment and treatment from an attachment theory perspective given the strong emphasis on the parent–child relationship. This view is supported by Buckmaster et al.’s (2019) review of family factors and self-injury, which found seven studies confirming a statistically significant relationship between a disorganized attachment style and NSSI and no studies that did not.

Given the typical age of onset of NSSI, particular attention is needed to change in the attachment relationship during adolescence. Attachment researchers often emphasize that adolescence is a time of significant change in the attachment relationship, a shift comparable in importance to when a toddler learns to walk (Ainsworth, 1989). In adolescence, this shift is often motivated by hormonal changes and the development of increasing independence. Open communication, accessibility, protection, and help are the most important factors for secure attachment in adolescence (Ainsworth, 1989). However, this is contrasted with the concealed nature of NSSI in many cases. Empirical research is needed, but it seems likely that the concealed nature of (most) NSSI would be highly relevant to the relationship between NSSI and having a disorganized attachment style (Buckmaster et al., 2019).

Hamadi and Fletcher (2021) suggest that children with intellectual disabilities are more likely to have disorganized attachment styles, yet there is limited research available. In a scoping review, Rinaldi et al. (2022) found challenging behavior in individuals with IDD is significantly linked to attachment difficulties. However, the seven studies included in the Rinaldi et al. review defined challenging behavior in different ways, and it was unclear whether SIB was included in all definitions, nor has its relationship with attachment difficulties been specifically examined. Since there is less research based on attachment theory in SIB than there is in NSSI, it is unclear if attachment is less relevant to SIB or if there is simply insufficient research.

##### Parenting behaviors

Parenting control is typically distinguished as psychological control or behavioral control. Psychological control refers to control over the child’s “psychological world (e.g., feelings, verbal expressions, identity, and attachment bonds)” while behavioral control refers to control over the child’s behavior (Barber, 2002, p. 4). In a large study of Flemish 12-year-olds (*n* = 1349), participants who engaged in NSSI perceived more parental behavioral and psychological control, though their parents did not report that they were significantly more controlling than other parents (Baetens et al., 2014). In another population, Canadian high schoolers who perceived their parents as less supportive of their autonomy engaged in more NSSI (partially mediated by difficulties with emotional regulation; Emery et al., 2017).

Overall, perception of greater parent support is associated with less NSSI (Bean et al., 2022). This support may take the form of a parent’s expressions of validation of an adolescent’s emotional experience or responsiveness to their self-injuring behaviors (if they are aware of them). Conversely, lack of support and invalidation are associated with greater NSSI and continued engagement in the behavior over time; in a longitudinal survey study, Chinese adolescents who perceived their families as invalidating of their emotional experience were more likely to still engage in self-injury a year after initial data collection (You & Leung, 2012). With an entirely different method, Crowell et al. (2013) confirmed an association between maternal invalidation and adolescent anger, opposition, and defiance, leading to emotional dysregulation that often resulted in self-injury for adolescents who already engaged in NSSI. They facilitated a conflict between mother–child dyads by providing purposefully incendiary discussion topics, then observationally coded these interactions with a microanalytic system and measured arousal with respiratory sinus arrhythmia (RSA) (Crowell et al., 2013; RSA is an index of heart rate variability and thought to reflect or relate to emotional regulation and its cognitive control). Increasing parental warmth is often a point of intervention in family-level interventions for adolescents who self-injure (Bean et al., 2022). This is intended to address the impacts of perceived invalidation and lack of support. Increased and consistent parental warmth along with behavioral support is associated with less engagement in NSSI (Baetens et al., 2014).

When a parent or caregiver has limited involvement with an adolescent, this increases the likelihood of NSSI (Brunner et al., 2014). This finding held up across populations from 11 European countries, suggesting cross-cultural generalizability, at least in Europe.

Parental criticism is a risk factor in NSSI (Bean et al., 2022). This is typically conceptualized in terms of affect regulation, such that criticism becomes internalized shame and this negative emotion is released through self-injuring behaviors (Amoss et al., 2016).

Chadwick et al. (2008) did not find an association between rates of expressed parental warmth and SIB in adolescents with severe IDD, though they did find higher rates of expressed parental criticism were associated with challenging behavior including SIB (though the association did not reach statistical significance). Woodman et al. (2015) found an association between maladaptive behaviors (including SIB) and praise, such that more praise in speech samples from the mother predicted fewer maladaptive behaviors in her adolescent or adult autistic child.

Two intervention studies on parenting and SIB have found statistically significant effects with different approaches. Singh et al. (2006) conducted an intervention study on mindful parenting. Three mothers of children with autism took a 12-week course on the philosophy and practice of mindfulness, and researchers found a significant decrease in their child’s challenging behavior, including self-injury, following the course (Singh et al., 2006). They interpreted this finding as evidence for the positive impacts of parenting behavior based in mindfulness and its components of unconditional acceptance, focusing on one thing at a time, and “reducing attempts by the mother to impose her will on her child” (Singh et al., 2006, p. 174).

Fodstad et al.’s (2018) intervention study focused on adaptive behaviorally-based training for parents of young children with IDD and SIB. Eleven parents completed an 11-week course designed to teach behavioral intervention techniques. Researchers found that parental use of maladaptive interaction strategies decreased significantly (such as intrusion on the child’s independence, inappropriate command, criticism, or lack of follow through; Fodstad et al., 2018). While this study is written in language more common to the field of ABA than the parenting behavior literature, it addresses similar elements of parent–child interaction in terms of how the parent responds to and initiates with the child.

#### Parent–parent

Parent–parent conflict is associated with increased risk of NSSI and suicidal behavior in adolescents (Baetens et al., 2014; Fortune et al., 2016). Otherwise, there is limited research on the parent subsystem and self-injury in two-parent families, that is, how the interactions between parents impact a child’s or adolescent’s self-injury (and vice versa). Under the framework of family systems theory, it is assumed that each subsystem influences the individuals in the family and the whole, as well as the whole and the individual influencing each subsystem.

Research dating back to the 1950s has established that parents of children with IDDs have higher divorce rates than parents of children without IDDs, though more recent research takes issue with the mass media conception that these marital relationships are “doomed for divorce” (Hartley et al., 2011, p. 2). In families experiencing IDD, increased child challenging behaviors is associated with lower marital satisfaction (though severity of IDD is not; Hartley et al., 2011). These researchers acknowledge the likely bidirectional nature of this relationship, in that marital quality may “indirectly impact children through altering parenting emotions and behaviors” (Hartley et al., 2011, p. 18). It is unclear in this study if challenging behavior includes SIB or if SIB impacts marital quality differently than other challenging behaviors. Overall, more research is needed to understand the parent–parent subsystem and self-injury.

#### Siblings

##### Birth order

There are mixed findings regarding a potential relationship between birth order and the likelihood of engaging in NSSI, but there is little research in this area. Kadric and Löfquist (2018) found no statistically significant effect of birth order on NSSI in young adults in Sweden. However, Kirkcaldy et al. (2009) found that middle children in Germany were more likely to engage in NSSI than firstborns, lastborns, or only children. In their study, NSSI was also correlated with the number of children in the family, such that individuals with more siblings were more likely to engage in NSSI (Kirkcaldy et al., 2009).

##### Relationship quality

Siblings of female adolescents who engage in NSSI reported feeling more coercion in their sibling relationship in Tschan et al.’s (2019) survey study (though the sibling with NSSI did not). The adolescents who engage in NSSI reported less warmth, empathy, similarity, and concern for boundary maintenance in their sibling relationship and higher rivalry, which Tschan et al. interpreted as evidence for parental favoritism.

##### Emotional impact

In the same study of siblings of female adolescents who engage in NSSI, siblings reported feeling sad, depressed, desperate, helpless, angry, scared, and guilty about their sister’s NSSI and experienced more internalizing symptoms (Tschan et al., 2019). Many felt that their sister’s behavior impacted the whole family (Tschan et al., 2019).

Siblings are more negatively impacted when their brother or sister with IDD has challenging behavior (Neece et al., 2010), and this is still the case for the subpopulation of siblings of individuals with severe or profound IDD (those most likely to engage in SIB; Roberts, 2021). Sibling’s coping, prosociality, and total difficulties are related to their brother or sister’s challenging behaviors; however, self-injury specifically has not been examined (Roberts, 2021).

The limited research available on siblings and both NSSI and SIB makes it difficult to draw conclusions about the sibling–sibling subsystem and self-injury. However, since challenging behavior appears to be an especially relevant variable for siblings of the individuals most likely to engage in SIB (those with more severe IDD; McClintock et al., 2003), it is worthwhile for researchers to consider the impact of SIB on siblings as well as how siblings may influence their brothers or sisters with IDD.

#### Overall summary of dyadic-level variables and self-injury

The majority of the literature at a dyadic level of the family system focuses on the parent and child in both NSSI and SIB. A major difference, however, is that attachment is a focus in the dyadic parent–child NSSI literature and is not in the SIB literature, despite evidence that disorganized attachment is common in children with IDD, especially those who engage in challenging behavior (Hamadi & Fletcher, 2021; Rinaldi et al., 2022). While parenting behavior, in a broad sense, is a focus for both literatures, a major limitation in the SIB literature is the lack of child-report of parenting behavior, which is particularly relevant given the differences found in the NSSI literature between parent and adolescent perceptions of parenting behavior. Both literatures have limited research on the parent–parent and sibling–sibling subsystems. Under the assumption that self-injury in one family member impacts all family members and all family members impact one family member’s self-injury (family systems theory), more research on parent–parent and sibling–sibling interactions is needed.

### Individual-level variables with potential bidirectional influence on the family

Next, we summarize research findings on individual-level variables and self-injury as they relate to the family context. While these variables are typically studied at the individual level, they have potential bidirectional influence when considered from a family systems lens. This section is not exhaustive but intended to give a sense of the types of individual-level variables researched in relation to SIB and NSSI and to highlight variables with the most relevance to the family system.

#### Child or adolescent variables

##### Sex differences

Recent research on sex differences has dispelled previous research indicating NSSI is more prevalent in females than males; rather, females are more likely to report self-injury and access clinical settings than males, and females and males tend to engage in different types of behaviors (females are more likely to cut, males more likely to burn or bang; Heath et al., in press).

##### Identity

In a psychosocial model of development, there is a tension during adolescence between identity confusion and identity synthesis (Erikson, 1968). During this period, adolescents work to establish a sense of personal identity and maintain consistency in their identity across settings. Identity confusion is significantly associated with engagement in NSSI (Claes et al., 2014) and identity confusion and synthesis mediate the association between maternal trust and alienation and engagement in NSSI (Gandhi et al., 2016). NSSI is linked to marginalized sexual orientation and/or gender identity, and transgender and nonbinary youth are especially likely to engage in NSSI (Speer et al., 2022).

##### Mental health

A meta-analytic study identified a list of predictors of engagement in NSSI, many of which relate to aspects of mental health: “in order of magnitude: prior NSSI, cluster b, hopelessness, prior suicidal thoughts and behaviors, exposure to peer NSSI, depressive symptoms, eating disorder pathology, being female, externalizing psychopathology, internalizing psychopathology, general psychopathology, and affect regulation” (Fox et al., 2015, p. 163; cluster b refers to diagnoses of antisocial, histrionic, narcissistic, and borderline personality disorders). In addition, children and adolescents prone to rumination are more likely to engage in self-injury, and Li et al. (2021) found that chronic rumination moderated the connection between interpersonal stress and NSSI. These researchers define rumination as a “passive and repetitive thinking tendency in which individuals dwell on thoughts about negative events and feelings” (Li et al., 2021, p. 1373). Rumination is a common symptom of anxiety (American Psychiatric Association, 2013).

Increased severity of intellectual or developmental disability, lower adaptive skills, expressive and receptive communication deficits, and a diagnosis of autism spectrum disorder are established individual risk factors for SIB (Dimian & Symons, 2022; McClintock et al., 2003). Engaging in stereotypy or restricted and repetitive behaviors, primary sensory deficits (deafness, blindness, or deafblindness), hyperactivity, and impulsivity are also associated with SIB (Dimian & Symons, 2022).

The salient individual difference variables in the NSSI and SIB literatures tend to be different, though both literatures examine features of diagnoses related to IDD or mental illness as well as comorbid diagnoses. The focus in the NSSI literature on the process of identity synthesis and marginalized identity status is not present in the SIB literature. Sex differences in the SIB literature are complicated by variations in the incidence of certain IDD diagnoses associated with SIB (e.g., Rett syndrome primarily affects girls and women), so are not typically included in discussions of risk for SIB.

#### Parent variables

Parents of children or adolescents engaging in NSSI report caregiving strain, defined as “adverse thoughts, feelings, and consequences experienced by those caring for someone who is suffering” (Curtis et al., 2018, p. 8). There is an association between a parent’s mental health, history of abuse, and self-criticism and their child’s engagement in NSSI; however, this should not be interpreted as a causal relationship nor does this association capture the full complexity of a family (in addition, self-injury was not distinguished by suicidality; Fortune et al., 2016). Parents coping with a child’s NSSI tend to feel socially isolated and may feel a need to be always available for their child, which sometimes conflicts with full-time employment (Ferrey et al., 2016).

In research on parents of children with IDD, self-reported stress is more strongly linked to a child’s challenging behavior than the severity of their developmental delay (Rojahn et al., 2008), such that child challenging behavior and parenting stress have a “mutually escalating effect on each other” (Baker et al., 2003, p. 227). Further, stress impacts a caregiver’s ability to implement interventions (Furniss & Biswas, 2020). Soke et al.’s (2016) secondary analysis of two major autism monitoring data sets found an association between lower maternal education and SIB, but no associations between SIB and parent age. Parent mental health has also been found to be negatively associated with a child’s challenging behaviors in a sample of mothers of children with fragile X syndrome (Fielding-Gebhardt et al., 2020). Again, it is important to acknowledge that this association does not indicate a causal relationship nor does it represent the complexity of a family.

A key distinction in the parent experience of self-injury is that parents tend to be aware if their child is engaging in SIB while they may not be aware of engagement in NSSI (Kelada et al., 2016; concealability is discussed in a later section). Otherwise, the limited research indicates similarities between parents of children engaging in SIB and NSSI, such as the relevance of parent mental health, educational status, and stress (or caregiving strain). However, some variables are more amenable to intervention than others. Parent variables which are amenable to intervention are more relevant to family-level interventions, such as parent self-criticism.

#### Overall summary of individual-level variables and self-injury

There is much more research available on individual-level variables in NSSI than SIB.

The variables that are studied in the individual with IDD and SIB tend to relate to the nature of the intellectual or developmental disability or comorbid diagnoses such as sensory deficits. There is limited research otherwise. NSSI researchers have studied specific features of diagnoses associated with NSSI as well as broader features of mental health and individual differences such as identity synthesis. Further, while SIB researchers have identified associations, such as the association between severity of intellectual impairment and SIB (McClintock et al., 2003), NSSI research has advanced to the level of sorting predictors by order of magnitude (Fox et al., 2015).

Parent variables indicate the immense strain of caring for children or adolescents with SIB or NSSI. Neither literature has significant research available on individual-level variables in parents, but according to family systems theory, variables at the dyadic level of the parent–child subsystem may hold more relevance to family-level interventions.

### Interdisciplinary knowledge translation: Applying NSSI research to SIB in IDD

More research is needed in a variety of areas related to SIB in IDD and families. Until that research is available, knowledge translation may inform interventions for families with children with IDD and SIB who urgently need support. We now delineate key differences between NSSI and SIB and explore four potential avenues of knowledge translation based on the previous review: family patterns according to the Circumplex Model, parenting behaviors, attachment between the parent and child, and the role of emotion in self-injury. These topics were chosen for initial focus because they are areas with significant research in NSSI, significant *lack* of research in SIB, and potential relevance to the development of family practitioner interventions for SIB in IDD. There are other areas of the NSSI literature that may be useful for SIB researchers (i.e., sibling impacts, the parent–parent subsystem), but it is not possible to address every topic area in this initial inquiry. As there is more research available on NSSI and the research question of this review pertains to SIB in IDD, we focus on how research on NSSI may be useful to advance research on SIB.

### Key differences between SIB and NSSI

When attempting knowledge translation and exchange between SIB and NSSI, recall primary differences between the two types of self-injury, notably, differences of form, concealability, and the individual’s capacity to self-report. SIB and NSSI usually present with different topographies and tend to differ structurally in terms of the intensity, frequency, and duration of the behavior(s). Most often, NSSI is an intense, infrequent, acute behavior (e.g., cutting on the wrists or thighs). SIB tends to be a restricted and repetitive behavior with greater frequency and duration and more variability in intensity (e.g., sometimes headbanging or hitting with low intensity yet repetition; at other times, a strike with enough force to cause retinal detachment). While SIB is most often an apparent behavior, NSSI is often hidden or concealed, even caregivers may be unaware that an adolescent is engaging in self-injury (Kelada et al., 2016). Finally, the ability to self-report, or describe one’s emotional state and reasons for engaging in self-injury, is often (though not always) absent in individuals who have severe IDD. Self-report is presumed in individuals who do not have IDD, and self-report is often relied upon in therapeutic interventions for NSSI.

### Key translational opportunities

#### Family patterns and SIB in IDD

The focus in the NSSI literature on patterns of family dynamics, such as family cohesion, communication, and flexibility, has informed the development of evidence-based family practitioner interventions (e.g., Schade, 2013; Wester & King, 2018). These interventions aim to promote healthy levels of cohesion in families (neither enmeshed nor disengaged), improve family communication, and promote flexibility. For example, Miner et al. (2016) describe the importance of “warm and flexible communication patterns” between adolescents and parents in structural family therapy and identify flexible communication as “vital to help the adolescent feel safe to explore the function of the NSSI within the family context” (p. 216).

Research indicates that cohesion, communication, and flexibility are important variables for families of individuals with IDD as well (Baker et al., 2011; Greeff & Nolting, 2013; Taylor et al., 2023), yet similar interventions for families in need of support for SIB in IDD have not been developed. Families experiencing SIB in IDD, especially chronic, entrenched cases, need support; family caregivers report enormous family stress (Roberts et al., 2023). It would therefore be of great clinical importance to adapt family practitioner interventions for use in families experiencing SIB.

Given the differences between SIB and NSSI, it is likely that interventions that work for families experiencing NSSI need to be adapted for families experiencing SIB, particularly with respect to the individual’s capacity for self-report. For example, there are numerous adaptations to individual psychotherapy for individuals with IDD that may be useful in family interventions; a review identified 94 adaptations in the research literature (Whitehouse et al., 2006). Flexibility in method is the most common adaptation to therapy for adults with ID. Other common adaptations include simplification, using plain language, and integrating activities at the individual’s developmental level (Whitehouse et al., 2006). The adaptive techniques that are appropriate will vary depending on the individual and their family. Regardless of how the individual with IDD is engaged in therapy, an advantage of interventions at the family level is that all family members are (ideally) engaged and contributing to therapeutic progress.

#### Parenting behavior and SIB in IDD

Another key area of the NSSI literature with relevance to the development of family-level interventions for SIB is parenting behavior. Parenting behavior is an important variable for understanding SIB as it is in NSSI, though some of the constructs related to parenting behavior in each literature differ (e.g., involvement or praise). Parenting behavior is also a variable that has successfully been a point of intervention to improve outcomes in both NSSI and SIB (Baetens et al., 2014; Singh et al., 2006). A key difference in this area, however, is that research in NSSI indicates there are often differences in perceptions of parenting behavior between the parent and the adolescent. Research comparing parent and child report is not available in SIB (see Key differences between SIB and NSSI and the issue of self-report).

Typically, NSSI research is conducted from a developmental psychology lens and SIB research is conducted from an ABA lens. These disciplines have different implicit assumptions, theoretical frames, and terminology. For example, Fodstad et al.’s (2018) intervention study is behaviorally-based and focuses on parent–child interactions in terms of reinforcement contingencies, while Baetens et al.’s (2014) study of parenting control cites Yates’ (2004) developmental model in which NSSI develops in a context of childhood abuse or neglect. These are two different ways to frame maladaptive childhood contexts that may lead to the development of self-injury.

While family practitioners may draw from principles of behavior analysis such as the idea of the function of a behavior, the conceptualization of most family-level interventions is more aligned with the developmental psychological lens (i.e., emotionally-focused or attachment-focused family therapy; Kissil, 2011; Schade, 2013). Thus, an area of knowledge translation may be to apply a developmental psychology lens to the understanding of parenting behaviors in SIB in IDD. For example, a reinforcement contingency in which a child’s communication attempt is ignored by a parent may be re-framed as an instance of parent invalidation (a parenting behavior variable associated with NSSI; Crowell et al., 2013). In another example, Fodstad et al.’s (2018) variable “intrusion on the child’s independence” (p. 3851) may be re-framed as parenting control (another parenting behavior associated with NSSI; Baetens et al., 2014). For a caregiver, this re-framing may facilitate the generalization of parenting behavior change. For professionals, this re-framing may facilitate interdisciplinary understanding and collaboration. Particularly with respect to the development of family-level interventions, applying a developmental psychology lens to existing literature on SIB in IDD may facilitate the application and adaptation of family therapy methods of intervention.

#### Attachment and SIB in IDD

In the NSSI literature and many family-level therapeutic interventions for NSSI, there is a focus on the attachment between parent and child (Buckmaster et al., 2019; Kissil, 2011). This emphasis is absent in SIB, yet children with IDD seem to be at increased risk of experiencing difficulties with attachment (Hamadi & Fletcher, 2021). On the parent side, potential factors in the development of attachment difficulties are stress, mental health problems, feelings of grief and loss, and struggling to identify their child’s needs (Hamadi & Fletcher, 2021). On the child side, some researchers have identified specific cognitive skills that may be necessary for secure attachment, such as connecting means (attachment signals) to an end (contact or proximity), or the role of impaired object permanence in separation distress for children with severe intellectual disabilities (Janssen et al., 2002). Further, children with IDD sometimes have a different physical response to their parent than children without IDD, and physical cues such as eye contact are believed to be important in the development of a healthy attachment between parent and child (Hamadi & Fletcher, 2021).

There are multiple family-level interventions based on attachment theory, which are used in clinical practice for the treatment of NSSI, including attachment-based family therapy (Kissil, 2011) and emotionally-focused family therapy (Schade, 2013). A stress-attachment model has been used to conceptualize other challenging behaviors in IDD research in which disorganized attachment, increased stress, and decreased use of coping strategies create risk of challenging behavior (Janssen et al., 2002). Given the importance of attachment theory to interventions in NSSI, it is worth exploring whether this model is useful for conceptualizing family interventions for SIB in IDD.

#### Emotion and SIB in IDD

Strong emotion as an antecedent to self-injury is an implicit assumption in the majority of the NSSI literature. Indeed, numerous models of NSSI emphasize emotional or affect regulation at multiple levels of the family system, including the cognitive emotional model, emotional cascade model, NSSI family distress cascade theory, and transactional models emphasizing invalidating family interactions (Hasking et al., 2017; Waals et al., 2018). Yet, emotion is rarely of primary consideration in the study or treatment of SIB in individuals with IDD, even though individuals with mild or moderate IDD self-report strong emotional experiences similar to the general population (Samways et al., 2022).

At the individual level, some research does exist on arousal and SIB, which, though it is not often explicitly discussed, is related to emotion and emotion regulation (e.g., Furniss & Biswas, 2020; Rojahn et al., 2008). In the core affect theory of emotion, arousal is conceptualized as a component of emotion along with valence (Russell, 2003). On a basic level, the core affect theory posits that emotional states, or *core affect*, can be plotted along axes of negative to positive valence and low to high arousal. For example, sadness is a low arousal-negative valence state, while anger is a high arousal-negative valence state. In a core affect theoretical context, arousal is a central component of emotional experience and arousal research is highly relevant to the discussion of emotion.

The research that is available on SIB and arousal tends to be conceptualized within a behavioral lens (e.g., Hoch et al., 2013), such that SIB is a conditioned response, which may regulate chronic over- or under-arousal. Understanding arousal from a different theoretical lens may be a first step toward knowledge exchange with NSSI research on emotion. For example, an NSSI researcher might argue that arousal regulation is also learned, to some extent, in the context of an early attachment relationship: “patterns of dyadic interaction and regulation in early development can entrain excitatory and inhibitory processes in the brain that underlie the child’s capacity for arousal modulation” (Kissil, 2011, p. 315). It is not clear how early experiences with caregivers impact a child’s ability to modulate their arousal, nor how this might relate to the development of SIB in children with IDD. It may be useful to integrate existing research indicating the role of arousal in SIB with the other dimension of emotion according to the core affect theory—that is, valence.

SIB research also gets close to emotion in research examining mental health variables such as anxiety. A study comparing boys with fragile X and Prader Willi syndromes found that anxiety associated with SIB was significantly more prevalent in the boys with fragile X syndrome, while the boys with Prader Willi tended to have tantrum outbursts reflecting, perhaps, syndrome-related patterns of behavior (Woodcock et al., 2009). Similar syndrome-related patterns of emotional behavior were not noted in the review of the NSSI literature. In NSSI, the emotional lens tends to be applied at the individual, dyadic, or family level rather than onto a particular population or diagnostic group.

As evident in this review, the NSSI literature frequently returns to the idea that NSSI is, for many, a form of coping with overwhelming emotions from negative life experiences or patterns of experience. This is seen in the numerous associations between NSSI and negative experiences and patterns, such as disengaged or enmeshed family cohesion, harmful relational patterns, abuse, trauma, divorce, disorganized attachment, coercive sibling relationships, and identity confusion (Buckmaster et al., 2021; Claes et al., 2014; Nixon et al., 2008; Peel-Wainwright et al., 2021; Tschan et al., 2019; Yates, 2004). There are challenges to the assessment of negative experiences such as trauma in individuals with IDD, but given the numerous findings linking NSSI to negative life experiences and patterns, it may be worthwhile for SIB researchers and clinicians to consider the conceptualization of SIB through the emotional regulation perspectives.

## LIMITATIONS

This was a selective conceptual rather than systematic review and did not purport to capture all publications available. Classifications and definitions of behavior (SIB, NSSI, and challenging behavior) are sometimes inconsistent in the literature, as is measurement of latent constructs such as family functioning. While this review intended to encompass an interdisciplinary viewpoint, it did not represent all disciplinary perspectives on self-injury, such as those of sociology, anthropology, or philosophy.

This review paper was limited by the breadth and state of the relevant literatures. There is very limited research available from a non-Western perspective or exploring ecological factors with relevance to self-injury beyond the family-level (e.g., cultural, societal, and political). Some work is emerging on NSSI and online communities/social media; this work could be expanded to other sociocultural or political factors with potential relevance to self-injury, such as mental health stigma and treatment access.

## CONCLUSION

In the dominant model of SIB research, the family is primarily a source of social reinforcement. In the dominant model of NSSI research, the family is primarily a source of socioemotional development. There are many practical reasons for why a research landscape like this has developed: SIB tends to start early, is observable, and is shown to increase in frequency for many according to contingent social attention. This creates an environment that lends itself to research focusing on micro-levels of parent–child interaction through a lens of behavioral theory. By contrast, NSSI tends to start later, is often unobservable in real time, and risk is informed by early experiences (such as abuse or neglect). These features of the phenomenon lend themselves to a developmental attachment lens.

Yet, the persistence of self-injury in clinical and community settings suggests that the historical methods of research and practice are insufficient. Particularly with regard to SIB in IDD, decades of research have led to progress related to etiology and treatment, but this review has documented that there are still many gaps in research in relation to developmental and family variables. We believe the history of siloed research and practice in the study and treatment of self-injury in individuals with and without intellectual and developmental disabilities has slowed the rate of progress by limiting perspectives.

One effective way forward may be continued interdisciplinary knowledge exchange. While family patterns, parenting behavior, attachment, and emotion are highlighted as key translational opportunities in this paper, they are not the only opportunities for research on NSSI to inform research on SIB. As intervention research in SIB in IDD is historically behaviorally focused (Rojahn et al., 2008), any consideration of the psychosocial elements with research-based associations in NSSI—at the family, dyadic, or individual level—would be a novel perspective that may lead to insights as to an individual’s interpersonal or emotional experience (regardless of their ability to articulate it). Researchers, and the systems in which they function, should continue to adapt and change to support interdisciplinary work for mutual benefit and ensure this work reaches communities in need of support for self-injury.

## Significance

“From a prevention perspective, features of the family environment may well be a more promising target for psychosocial intervention than the individual risk factors identified to date” (Furniss & Biswas, 2020, p. 41). There is an urgent need to support individuals who have intellectual and developmental disabilities and engage in SIB. The family is often the individual’s greatest support and closest community, and they, too, are vulnerable to the negative impacts of their family member’s self-injury. Interdisciplinary, collaborative, and innovative approaches hold promise to improve support for families.
